# Replication-Coupled PCNA Unloading by the Elg1 Complex Occurs Genome-wide and Requires Okazaki Fragment Ligation

**DOI:** 10.1016/j.celrep.2015.06.066

**Published:** 2015-07-23

**Authors:** Takashi Kubota, Yuki Katou, Ryuichiro Nakato, Katsuhiko Shirahige, Anne D. Donaldson

**Affiliations:** 1 Institute of Medical Sciences, University of Aberdeen, Foresterhill, Aberdeen AB25 2ZD, Scotland, UK; 2 Research Center for Epigenetic Disease, Institute of Molecular and Cellular Biosciences, The University of Tokyo, Tokyo 113-0032, Japan

## Abstract

The sliding clamp PCNA is a crucial component of the DNA replication machinery. Timely PCNA loading and unloading are central for genome integrity and must be strictly coordinated with other DNA processing steps during replication. Here, we show that the *S. cerevisiae* Elg1 replication factor C-like complex (Elg1-RLC) unloads PCNA genome-wide following Okazaki fragment ligation. In the absence of Elg1, PCNA is retained on chromosomes in the wake of replication forks, rather than at specific sites. Degradation of the Okazaki fragment ligase Cdc9 leads to PCNA accumulation on chromatin, similar to the accumulation caused by lack of Elg1. We demonstrate that Okazaki fragment ligation is the critical prerequisite for PCNA unloading, since *Chlorella* virus DNA ligase can substitute for Cdc9 in yeast and simultaneously promotes PCNA unloading. Our results suggest that Elg1-RLC acts as a general PCNA unloader and is dependent upon DNA ligation during chromosome replication.

## Highlights

• PCNA is retained behind all replication forks that lack Elg1
• Elg1-RLC cannot unload PCNA from unligated lagging-strand DNA
• An exogenous ligase can join Okazaki fragments to promote PCNA unloading
• Elg1-RLC acts as a general PCNA unloader, dependent upon Okazaki fragment ligation

## Graphical Abstract

**Figure:**
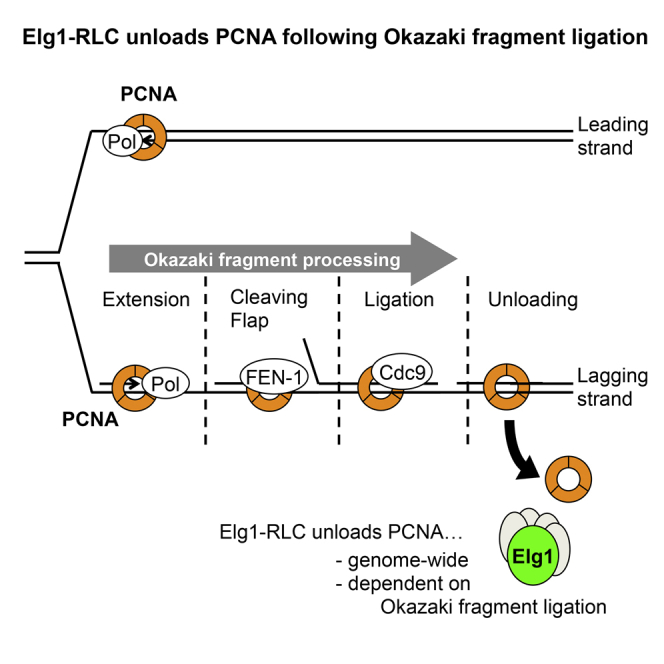

## Teaser

The sliding clamp PCNA is crucial to DNA replication fidelity, and loading and unloading of PCNA must be strictly regulated. Kubota et al. find that the Elg1 complex unloads PCNA behind all replication forks and is dependent upon successful DNA ligation of Okazaki fragments on the lagging strand.

## Introduction

Integrity of the DNA replication machinery is crucial to ensure accurate duplication of the genetic information and subsequent transfer to daughter cells. The ring-shaped homotrimeric protein PCNA (proliferating cell nuclear antigen) has a central role in DNA replication, coordinating the action of many replisome-associated proteins (Krishna et al., 1994). PCNA encircles DNA to act as a sliding clamp, ensuring processivity of DNA polymerases, and a platform for recruitment of numerous other replication proteins (Moldovan et al., 2007). Two important components whose recruitment is assisted by direct interaction with PCNA are the flap endonuclease FEN-1 and DNA ligase I (Beattie and Bell, 2011). Both these proteins are involved in the processing of Okazaki fragments, the series of short fragment precursors first synthesized and then ligated to assemble the nascent lagging strand. On the lagging strand, PCNA must be loaded on the DNA repeatedly, at the initiation of each Okazaki fragment. PCNA is loaded onto primer-template junctions by replication factor C (RFC), a hetero-pentameric complex consisting of one large subunit, Rfc1, and four smaller ones, Rfc2–5 (Bowman et al., 2004; Gomes and Burgers, 2001; Kelch et al., 2011). After completion of each Okazaki fragment, PCNA is believed to be unloaded from DNA and recycled to promote fidelity of synthesis of subsequent Okazaki fragments.

The Elg1 RFC-like complex (Elg1-RLC), in which Elg1 replaces Rfc1 to associate with Rfc2–5, acts in DNA replication (Kanellis et al., 2003). Previous results indicate one probable molecular function of *S. cerevisiae* Elg1-RLC is unloading of PCNA during DNA replication (Kubota et al., 2013a, 2013b). The function of the Elg1-RLC in PCNA unloading appears to be conserved in humans, since ATAD5 (the human Elg1 homolog) is required for proper removal of PCNA from chromatin in human cell lines (Lee et al., 2013; Shiomi and Nishitani, 2013).

When DNA synthesis is blocked, PCNA becomes mono-ubiquitinated at K164 to promote polymerase exchange, which enables DNA repair (Bienko et al., 2005; Hoege et al., 2002). In contrast, SUMOylation of PCNA (at K164 and K127) is stimulated simply by association with DNA and occurs during S phase even in the absence of exogenous damage (Hoege et al., 2002; Parker et al., 2008). One role for PCNA SUMOylation appears to be recruitment of the antirecombinogenic helicase Srs2 to prevent inappropriate recombination (Armstrong et al., 2012; Papouli et al., 2005; Pfander et al., 2005). Elg1-RLC preferentially binds SUMOylated PCNA, although SUMOylation of PCNA is not necessary for its unloading by Elg1-RLC (Kubota et al., 2013b; Parnas et al., 2010).

Loss of yeast Elg1 causes genome instability including gross chromosomal rearrangements, increased spontaneous sister chromatid recombination, defective sister chromatid cohesion, and derailed telomere length maintenance (Bellaoui et al., 2003; Ben-Aroya et al., 2003; Kanellis et al., 2003; Maradeo and Skibbens, 2009; Parnas et al., 2009; Smolikov et al., 2004). This requirement for Elg1 for genome maintenance seems to be conserved in higher eukaryotes, since mice with reduced expression of ATAD5 (the mammalian Elg1 ortholog) show genome instability and develop tumors (Bell et al., 2011). Elg1 is therefore crucial for genome maintenance.

Where and how the Elg1-RLC ensures timely unloading of PCNA from chromatin has until now remained obscure. In particular, the defective sister chromatid cohesion and derailed telomere length maintenance observed in *elg1*Δ yeast strains raised the possibility that Elg1-RLC unloading function might be limited to particular locations such as cohesion sites or chromosome ends. Moreover, it was unclear whether any interdependence exists between PCNA unloading and Okazaki fragment processing. Here, we show that the Elg1-RLC unloads PCNA genome-wide, rather than at specific chromosomal sites. By dissecting DNA ligase functions and examining effects of substituting yeast DNA ligase with an exogenous viral ligase, we find moreover that unloading of PCNA by Elg1-RLC depends on completion of Okazaki fragment ligation. Our results indicate that the Elg1-RLC acts as a general PCNA unloader on the lagging strand, and its action depends on successful strand maturation.

## Results

### Simultaneous Expression of myc-Tagged PCNA and Untagged PCNA Permits Normal Cell Growth

**Figure 1:**
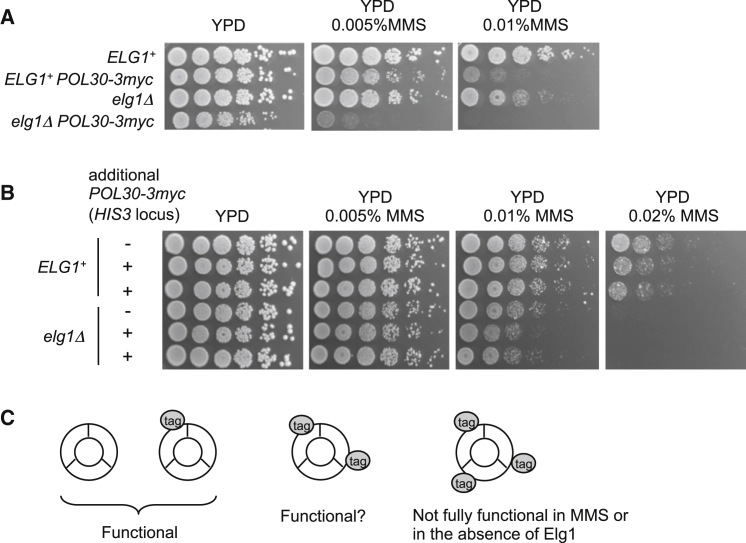
Expression of myc Tagged with Untagged PCNA Permits Normal Cell Growth (A) Cells with *POL30-3myc* as the only PCNA allele show sensitivity to MMS and, in an *elg1Δ* background, defective growth and hyper-sensitivity to MMS. Five-fold serial dilutions of cells were plated on YPD or YPD plus MMS and incubated for 2 days at 30°C. (B) Expression of myc-tagged PCNA in addition to endogenous untagged PCNA does not cause sensitivity to MMS or slow growth in the absence of Elg1. Five-fold serial dilutions of cells were plated on YPD or YPD plus MMS, and incubated for 2–3 days at 30°C. Two different isolates of *ELG1*^*+*^ and *elg1Δ* strains carrying *POL30-3myc* are shown. (C) Inferred functionality of PCNA trimers consisting of untagged and myc-tagged subunits. See also Figure S1.

PCNA accumulates on chromatin in the absence of Elg1 (Kubota et al., 2013b; Parnas et al., 2010). To investigate where on chromosomes PCNA accumulates in *S. cerevisiae* lacking Elg1, we carried out chromatin immunoprecipitation sequencing (ChIP-seq) analysis. As no ChIP-grade PCNA antibody is commercially available, we constructed strains with myc-tagged PCNA (*POL30-3myc*). An *elg1*Δ mutant carrying *POL30-3myc* as its only PCNA allele shows slow growth, when compared to *elg1*Δ with untagged PCNA (Figure 1A, left plate), indicating that myc-tagged PCNA is not fully functional in the absence of Elg1. Moreover, this myc-tagged PCNA allele caused increased sensitivity to the DNA-alkylating drug methyl methanesulfonate (MMS) in a wild-type background and extreme sensitivity to MMS in the absence of Elg1 (Figure 1A, middle and right plates). Similarly, FLAG tagging of PCNA at either the N terminus or C terminus compromised its function (Figure S1) (Davidson et al., 2012). We conclude that a PCNA trimer with all three subunits tagged cannot fulfill all normal PCNA functions, and strains carrying only a tagged version are not suitable to test the localization of PCNA.

To address this issue, we instead constructed strains with the *POL30-3myc* gene (under control of the native *POL30* gene promoter) inserted as a second copy in addition to endogenous untagged *POL30* gene. Insertion of tagged PCNA as a second copy in this way did not cause slow growth in the absence of Elg1, nor was sensitivity to MMS significantly increased (Figure 1B). Western blot analysis showed that both untagged and myc-tagged PCNA are loaded and, in an *elg1*Δ mutant, accumulate on chromatin (Figure S1C). SUMOylated forms of both untagged and myc-tagged PCNA were also increased, reflecting PCNA accumulation on chromatin in the *elg1Δ* mutant (Kubota et al., 2013b). These results suggest that a PCNA trimer containing both untagged and myc-tagged PCNA retains functionality (Figure 1C) and is suitable for ChIP-seq analysis using an anti-myc antibody.

### In the Absence of Elg1, PCNA Is Retained on Replicated DNA Genome-wide

**Figure 2:**
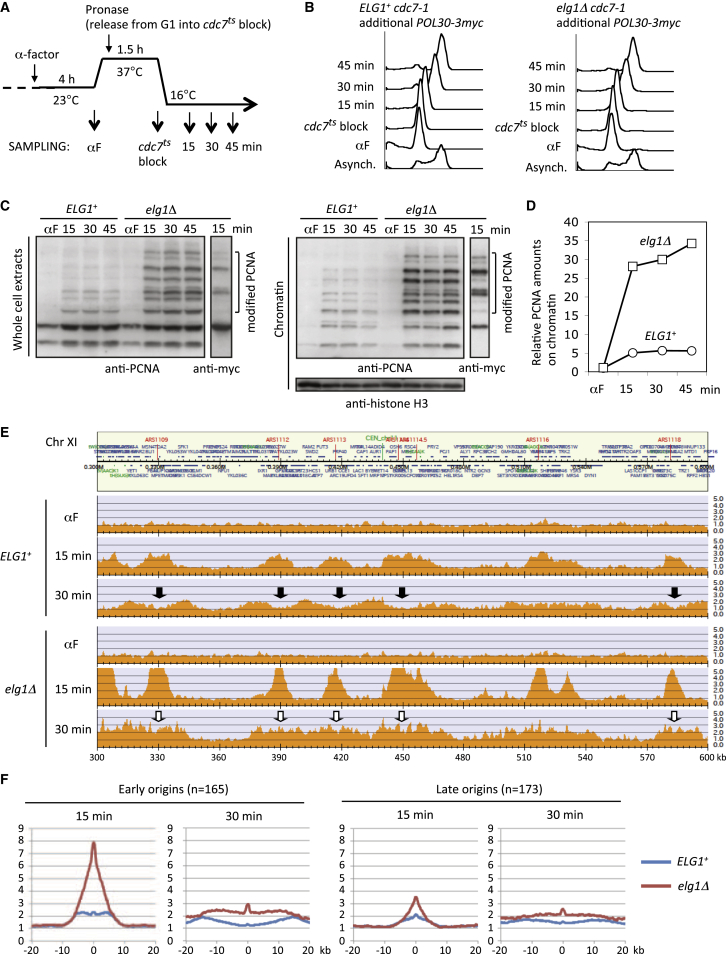
PCNA Is Retained on Replicated DNA Genome-wide in the Absence of Elg1 (A) Outline of procedure for chromatin fractionation and ChIP-seq experiments. (B) Flow cytometry analysis. (C) PCNA accumulates on chromatin in the absence of Elg1 during DNA replication. Myc-tagged and untagged PCNA in whole-cell extracts (left) and chromatin fractions (right) detected by western blotting with anti-PCNA antibody. (D) Quantification of total PCNA on chromatin in (C), expressed as relative increase compared to alpha-factor arrested cells. (E) ChIP-seq analysis of PCNA distribution on chromatin. PCNA is unloaded behind replication forks in *ELG1*^*+*^ (black arrows) but retained in *elg1*Δ (white arrows). The chromosome (Chr) XI 300–600 kb region is shown; the entire chromosome XI is presented in Figure S3. (F) Median PCNA enrichment around early (n = 165) and late (n = 173) origins at 15- and 30-min time points. See also Figures S2 and S3.

To synchronize replication fork movements between cells and examine where PCNA accumulates in the *elg1*Δ mutant, we utilized the *cdc7-1* temperature-sensitive mutation. *CDC7* encodes the catalytic subunit of Dbf4-dependent kinase (DDK) required for replication origin initiation (Jackson et al., 1993), and cells released from a *cdc7-1* block traverse S phase very synchronously (Figures S2A and S2B). Expression of additional myc-tagged PCNA in *cdc7-1* or *cdc7-1 elg1*Δ cells does not affect growth or S phase progression (Figures S2C and S2D). We confirmed that in *elg1*Δ mutant cells released into S phase at 16°C, both untagged PCNA and myc-tagged PCNA accumulate on chromatin (Figures 2A–2D, S2E, and S2F). ChIP-seq analysis showed that, in the presence of Elg1, PCNA is loaded close to replication origins in early S phase (15 min) (Figures 2E and S3). By mid-S phase (30 min post-release), PCNA is maximally associated with sites 10–15 kb away from origins, with PCNA having been unloaded behind the replication forks (dips in the 30 min time plots, black arrows; Figure 2E). In the absence of Elg1, PCNA in contrast accumulates on replicating regions (higher peaks within the origin regions in the 15 min time point, compared to *ELG1*^+^ strain) and is retained, without the appearance of the dips that in *ELG1*^+^ are indicative of timely PCNA removal (white arrows in the 30-min time point) (Figure 2E). These results imply that in the absence of Elg1 the association of PCNA with chromatin is prolonged following replication fork passage, probably due to a delay in unloading. PCNA signals in the *elg1*Δ mutant are reduced at 30 min when compared to the 15-min time point (Figure 2E), consistent with the previous finding that PCNA is unloaded eventually even in the absence of Elg1 (Kubota et al., 2013b). At the 45-min time point, clear PCNA peaks were no longer visible, probably due to asynchrony of replication forks. A similar pattern was seen genome-wide, with peaks of PCNA association with chromatin progressing outward from origin sites (chromosome XI is shown in Figure S3; genome-wide data can be found at http://www.iam.u-tokyo.ac.jp/chromosomeinformatics/Supplementary%20Material.html).

We suspect the very high PCNA signals at origin sites in the 15-min time point in the *elg1*Δ mutant, compared to *ELG1*^+^, reflect delayed unloading of PCNA (as opposed to PCNA over-loading at replication initiation), since PCNA unloading will begin as soon as lagging-strand replication is established and will therefore already have begun to occur at the 15-min time point in *ELG1*^+^ cells. This pattern was confirmed by a meta-analysis of median levels of PCNA around replication origins (Figure 2F). In the *elg1*Δ mutant, elevated levels consistent with delayed PCNA unloading were observed in the vicinity of early origins (n = 165) at both the 15- and 30-min time points, when compared to *ELG1*^+^ cells. A similar pattern was also observed around late origins (n = 173), but with reduced peak heights that probably reflect a lesser degree of synchrony between cells in the firing of late origins (Figure 2F). Interestingly, while PCNA signals generally diverged outward from origins as expected in the 15- to 30-min interval, small spikes of PCNA signal persisted at the actual origin sites (0 kb in Figure 2F) in both *ELG1*^+^ and *elg1*Δ mutant. These peaks might potentially indicate some specific difficulty in PCNA unloading following the replication initiation process or, alternatively, may reflect ongoing activation events caused by asynchrony of replication initiation in a minor fraction of cells in the population.

Other than around replication origins, there were no genome loci showing particularly excessive PCNA accumulation that might indicate retention of PCNA at specific chromosome sites (Figure S3; data are available at http://www.iam.u-tokyo.ac.jp/chromosomeinformatics/Supplementary%20Material.html). Overall, these results support the idea that Elg1-RLC unloads PCNA genome-wide, rather than at specific chromosomal sites.

### PCNA Accumulates on Chromatin in the Absence of the Replicative DNA Ligase Cdc9

**Figure 3:**
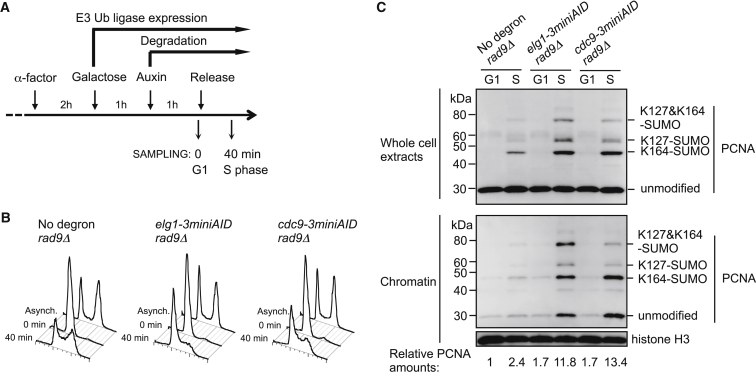
PCNA Accumulates on Chromatin in the Absence of the Replicative DNA Ligase Cdc9 (A) Outline of procedure for cell synchronization, induction of degradation of AID-tagged proteins, and sampling for chromatin fractionation. (B) Flow cytometry analysis. (C) In the absence of Elg1 (lane 4) or the DNA ligase Cdc9 (lane 6), PCNA accumulates on chromatin during DNA replication. Histone H3 is loading control. PCNA amounts on chromatin are indicated below, relative to no degron *rad9*Δ G1 sample (lane 1). See also Figure S4.

Our ChIP analysis suggested that the Elg1-RLC is a general replication-coupled PCNA unloader that prevents PCNA accumulation behind replication forks. We next tested at which step in DNA synthesis the Elg1-RLC unloads PCNA. PCNA SUMOylation is increased in cells in which the replicative DNA ligase Cdc9 is degraded (Kubota et al., 2013b), suggesting that PCNA may accumulate on chromatin in the absence of Cdc9. Since the primary role of Cdc9 in replication is ligation of lagging-strand Okazaki fragments, this observation raised the possibility that PCNA unloading might be coupled to Okazaki fragment ligation. We therefore tested directly whether PCNA accumulates on chromatin when Okazaki fragment ligation is blocked by degradation of Cdc9. We used a *cdc9-3miniAID* construct (*CDC9* tagged with three repeats of mini-auxin-inducible degron), allowing induced Cdc9 degradation upon auxin addition (Kubota et al., 2013b; Nishimura et al., 2009). We also deleted the DNA damage checkpoint mediator *RAD9* as described previously (Smith and Whitehouse, 2012), although deletion of *RAD9* is not strictly necessary for replication to proceed in ligase-depleted cells (McGuffee et al., 2013) and in later experiments *RAD9* was left intact unless indicated. *cdc9-3miniAID rad9Δ* cells were synchronized in G1 phase and degradation of Cdc9 induced prior to releasing the cells into S phase (Figures 3A and 3B). Western blot analysis of chromatin fractions (Figure 3C) revealed that PCNA does accumulate on chromatin during S phase in the absence of Cdc9, similar to the accumulation occurring in cells depleted of Elg1. These results led us to test the possibility that Elg1-RLC unloads PCNA following Okazaki fragment ligation.

When Cdc9 was degraded, we observed PCNA SUMOylation at K127 and K164. Under our experimental conditions, we did not observe modification of PCNA at K107 (Figure S4), although K107 ubiquitination has previously been reported in a *cdc9-1* temperature-sensitive mutant (Das-Bradoo et al., 2010).

### PCNA Retention on Chromatin with Unligated Okazaki Fragments Is due to Failure of Elg1-Dependent PCNA Unloading, Rather Than Re-loading of PCNA on Unligated Okazaki Fragments

**Figure 4:**
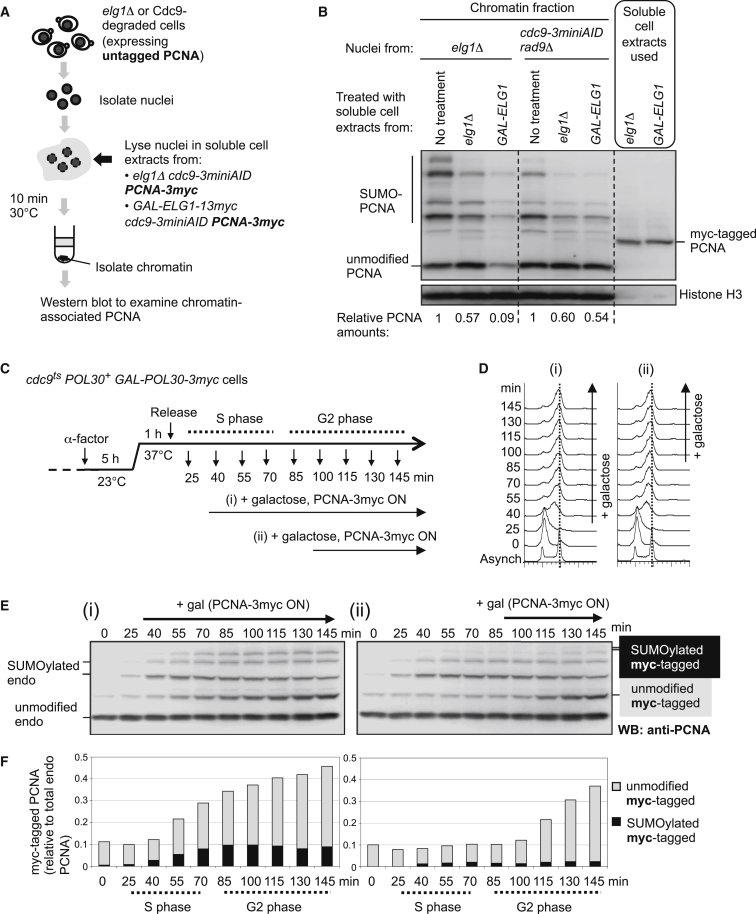
PCNA Retention on Chromatin following Cdc9 Degradation Is Caused by Failure of Elg1-Dependent PCNA Unloading, Rather Than Re-loading of PCNA on Unligated Okazaki Fragments (A) Outline of in vitro PCNA unloading assay. Soluble cell extracts contain myc-tagged PCNA to enable monitoring of any re-loading on chromatin. Cdc9 was degraded in cells used for soluble cell extracts to prevent DNA ligation during unloading assay. (B) In vitro assay shows that PCNA is not unloaded by Elg1 in the absence of DNA ligase Cdc9, and PCNA accumulation on chromatin is not due to re-loading. PCNA retained on chromatin after treating with indicated soluble cell extracts was detected by western blotting with anti-PCNA antibody. Analysis of soluble cell extracts used (last two lanes) confirmed presence of myc-tagged PCNA. Histone H3 is loading control for chromatin samples. PCNA amounts on chromatin indicated below, relative to no treatment. (C) Outline of experiments to test PCNA re-loading on unligated Okazaki fragments in vivo. Strain is the *cdc9-1* temperature-sensitive mutant with myc-tagged *POL30* gene as a second copy, expressed from the *GAL* promoter. Cells were arrested in G1 phase at 23°C, and cultures were shifted to a restrictive temperature of 37°C for 1 hr before release into S phase. Galactose was added to the culture 26 min (i) or 86 min (ii) after release. (D) Flow cytometry analysis. (E) No re-loading of PCNA takes place on unligated Okazaki fragments in vivo, as evidenced by lack of loading of myc-tagged PCNA expressed in G2 phase in the absence of Cdc9 (right panel). Myc-tagged PCNA is loaded if expressed in S phase in the absence of Cdc9 (left panel). Loading of myc-tagged PCNA on chromatin assessed by appearance of SUMOylated forms in whole-cell extracts. WB, western blotting. (F) Quantification of the anti-PCNA blots in (E). Amounts of unmodified PCNA-3myc (gray bars) or SUMOylated PCNA-3myc (black bars, reflecting chromatin bound) shown relative to total endogenous PCNA. See also Figure S5.

We envisaged two possible mechanisms that might cause the PCNA accumulation on chromatin in cells lacking Cdc9. First, Elg1-RLC may be unable to unload PCNA prior to ligation of Okazaki fragments. A second possibility is that the Elg1-RLC does unload PCNA from unligated Okazaki fragments, but PCNA is immediately re-loaded by RFC onto the nicked DNA, because RFC has been shown to load PCNA onto nicked DNA in vitro (Bylund and Burgers, 2005; Cai et al., 1996). To distinguish these possibilities, we first performed an in vitro PCNA unloading assay designed to test also for PCNA exchange using untagged and myc-tagged PCNA (Figure 4A). In this unloading assay, we prepared nuclei from *elg1Δ* mutant or Cdc9-degraded cells in S phase (both of which have untagged PCNA retained on chromatin). These nuclei were treated with extracts made from PCNA-3myc cells either containing or lacking Elg1. We then tested if the untagged PCNA derived from the nuclei was unloaded from chromatin (Figure 4A). When treated with cell lysate from cells overexpressing Elg1, the untagged PCNA from the *elg1*Δ mutants was unloaded (Figure 4B, third lane), but untagged PCNA on chromatin from the Cdc9-degraded cells was not unloaded (Figure 4B, sixth lane). We did not observe PCNA exchange, i.e., re-loading of extract-derived myc-tagged PCNA (Figure 4B, sixth lane). This result indicates that Elg1-RLC cannot unload PCNA from DNA assembled in the absence of the DNA ligase Cdc9 and that the retention of PCNA on chromatin in the Cdc9-degraded cells (Figure 4B, sixth lane) is not caused by in vitro re-loading by RFC on nicked DNA.

We also tested in vivo for re-loading of myc-tagged PCNA onto chromatin in cells lacking Cdc9 (Figures 4C–4F). To check whether PCNA is re-loaded continuously on unligated Okazaki fragments, we induced expression of myc-tagged PCNA in S or G2 phase in the absence of Cdc9 (Figures 4C and 4D) and monitored PCNA loading (assessed by the appearance of PCNA-3myc SUMOylated forms in whole cell extracts; Figures 4E, 4F, and S5). As expected, when expression of myc-tagged PCNA is induced in S phase, it is loaded onto chromatin, presumably associated with ongoing Okazaki fragment synthesis. However, PCNA-3myc is not loaded on chromatin when its expression is induced in G2 phase, when no new Okazaki fragments are being formed (although unligated DNA is still present as confirmed below). These results indicate that PCNA is not re-loaded continuously at unligated Okazaki fragments in vivo, supporting our interpretation that PCNA accumulation in cells lacking Cdc9 is due to failure of PCNA unloading by Elg1-RLC, rather than continuous re-loading of PCNA by RFC.

### Lack of DNA Ligase Activity of Cdc9, Not Lack of Its Interaction with PCNA, Causes PCNA Accumulation on Chromatin

**Figure 5:**
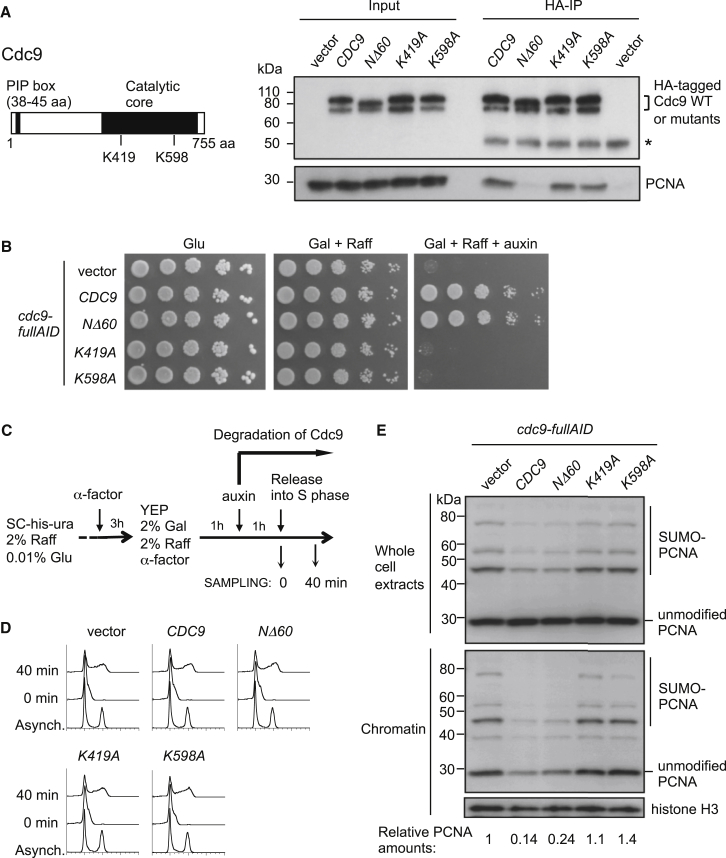
PCNA Accumulation on Chromatin Is Caused by Failure of DNA Ligation Rather Than Lack of Cdc9 Interaction with PCNA (A) Schematic structure of Cdc9 protein (left) and defective PCNA interaction of an N-terminally truncated Cdc9 mutant (*cdc9-NΔ60*) that lacks the PCNA interaction motif (right). HA-tagged versions of wild-type *CDC9* or its *NΔ60*, *K419A*, *K598A* mutants were overexpressed under the *GAL* promoter and immunoprecipitated with anti-HA antibody. PCNA was then detected by western blot with anti-PCNA antibody. Asterisk indicates a heavy chain of antibody used for immunoprecipitation. (B) *cdc9-NΔ60* complements the growth defect of cells with endogenous Cdc9 degraded. Serial dilutions (1:5) of cells grown on SC-His-Ura containing 2% glucose, 2% galactose + 2% raffinose, or 2% galactose + 2% raffinose + 0.5 mM auxin (IAA) for 2 days at 30°C. (C) Outline of cell synchronization, induction of degradation of endogenous Cdc9, and sampling for chromatin fraction. Plasmid-borne *CDC9* alleles being tested were expressed under the natural *CDC9* promoter from the *HIS3* marker plasmid pRS313. (D) Flow cytometry analysis. (E) PCNA accumulates on chromatin in *cdc9* ligase-null mutants (*cdc9-K419A* and *cdc9-K598A*), but not in a *cdc9* mutant defective for PCNA interaction (*cdc9-NΔ60*). PCNA in whole-cell extracts (top) and chromatin fractions (bottom) detected by western blotting with anti-PCNA antibody. PCNA amounts on chromatin shown below, relative to *cdc9-fullAID* with empty vector (vector). Histone H3 is the loading control.

Our data suggest that Okazaki fragment ligation by Cdc9 is required for PCNA unloading by the Elg1-RLC (Figure 4). We considered other possible explanations: (1) that Cdc9-bound PCNA is the target for unloading, or (2) that Cdc9 protein itself stimulates PCNA unloading, but not via its ligase activity. To address these possibilities, we tested whether mutant Cdc9 proteins disabled for different functions cause PCNA accumulation on chromatin. We used *cdc9-NΔ60* (which lacks the PIP [PCNA-interacting peptide] motif; see Figure 5A cartoon) and two ligase-null mutants, *cdc9-K419A* and *cdc9-K598A* (mutating the active center). Since yeast Cdc9 might contain PCNA-interacting sequences outside the PIP box (as is true for human DNA ligase I; Song et al., 2009), we first tested if Cdc9-NΔ60 binds PCNA. As expected, Cdc9-NΔ60 shows greatly reduced affinity for PCNA, but the two ligase-null mutant Cdc9 proteins were still able to interact with PCNA (Figure 5A). Despite its severely compromised PCNA interaction, expression of *cdc9-NΔ60* under the *CDC9* native promoter from a centromeric plasmid complements the growth defect of cells in which endogenous Cdc9 was degraded (Figure 5B), consistent with the previously reported complementation of a *cdc9-1 ts* mutant by this allele (Nguyen et al., 2013). In contrast, the two ligase-null mutants *cdc9-K419A* and *cdc9-K598A* failed to complement the growth defect of a Cdc9-degraded strain (Figure 5B) as expected, because DNA ligation is the essential function of Cdc9.

To test whether the Cdc9 mutant proteins can stimulate PCNA removal from chromatin, we induced degradation of the wild-type Cdc9 in cells expressing *cdc9-NΔ60*, *cdc9-K419A*, or *cdc9-K598A* prior to release into S phase (Figures 5C and 5D). Cells were harvested in mid-S phase and chromatin-bound PCNA examined (Figure 5E). Expression of wild-type Cdc9 or the PCNA interaction-defective mutant (*cdc9-NΔ60*) stimulated PCNA removal from chromatin, while expression of the two ligase-null mutants did not (Figure 5E). These results indicate that Cdc9 promotes PCNA unloading by Okazaki fragment ligation, rather than through its interaction with PCNA or a different Cdc9 function unrelated to ligation.

### Okazaki Fragment Ligation by *Chlorella* Virus DNA Ligase Facilitates PCNA Unloading

**Figure 6:**
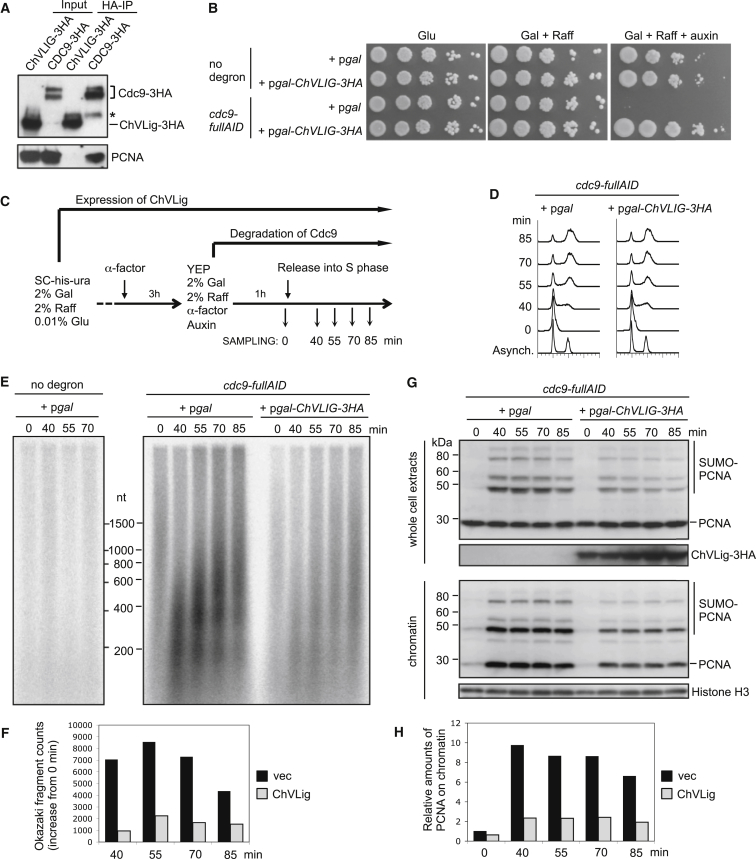
Okazaki Fragment Ligation by *Chlorella* Virus DNA Ligase Facilitates PCNA Unloading (A) *Chlorella* virus DNA ligase (ChVLig) does not physically interact with PCNA. HA-tagged ChVLig and Cdc9 overexpressed from the *GAL* promoter were immunoprecipitated with anti-HA antibody. PCNA was detected by western blot with anti-PCNA antibody. Asterisk indicates a heavy chain of the antibody used for immunoprecipitation. (B) *Chlorella* virus DNA ligase (ChVLig) complements the growth defect of cells in which yeast DNA ligase Cdc9 is degraded. Serial dilution (1:5) of cells grown on SC-His-Ura containing 2% glucose, 2% galactose + 2% raffinose, or 2% galactose + 2% raffinose + 0.5 mM auxin (IAA) for 2–3 days at 30°C. ChVLig is expressed under the *GAL* promoter. (C) Experimental outline for Okazaki fragment detection and chromatin fractionation. Galactose was added before cell-cycle synchronization to maximize expression of ChVLig. (D) Flow cytometry analysis. (E) Okazaki fragments can be ligated by ChVLig. Each lane contains genomic DNA prepared from same number of cells, with Okazaki fragments visualized by ^32^P end-labeling. nt, nucleotides. (F) Quantification of Okazaki fragments prepared from *cdc9-fullAID* carrying empty vector (vec) or expressing ChVLig (ChVLig) in (D). Values plotted are the increase over 0 min in intensity of fragments shorter than 1,500 nt. (G) PCNA retention on chromatin caused by Cdc9 degradation is partially relieved by expression of ChVLig. PCNA in whole-cell extracts (top) and chromatin fractions (bottom) detected by western blotting with anti-PCNA antibody. HA-tagged ChVLig expression was confirmed by anti-HA antibody. Histone H3 is the loading control. (H) Quantification of chromatin-bound PCNA in (F), expressed relative to vector 0-min sample. See also Figure S6.

If Okazaki fragment ligation is a central requirement to promote PCNA unloading (as opposed to the presence of Cdc9 protein itself), then Okazaki fragment ligation by an exogenous DNA ligase should also allow PCNA to be unloaded. To test this hypothesis, we used *Chlorella* virus DNA ligase (ChVLig), which can complement the growth defects of a *cdc9-1 ts* mutant or a *cdc9*Δ deletion mutant (Nguyen et al., 2013; Sriskanda et al., 1999). ChVLig is the smallest known ATP-dependent DNA ligase, containing only a conserved catalytic core (Ho et al., 1997). As ChVLig has no additional domains beyond this catalytic core, it was inferred that ChVLig is unlikely to interact physically with the eukaryotic replication proteins (Sriskanda et al., 1999). Co-immunoprecipitation analysis confirmed that ChVLig does not physically interact with PCNA, under conditions in which Cdc9 does (Figure 6A).

We confirmed that overexpression of ChVLig complements the growth defect of cells in which Cdc9 has been degraded (Figure 6B). Next, we examined whether Okazaki fragments are in fact ligated by ChVLig in the Cdc9-degraded cells (Figures 6C–6F). Okazaki fragments accumulated in Cdc9-degraded cells carrying empty vector, but levels were reduced when ChVLig is overexpressed (Figures 6E and 6F), indicating that ChVLig can ligate Okazaki fragments in yeast cells, although not as efficiently as endogenous Cdc9 (Figures 6E and S6). We then tested if PCNA is unloaded following Okazaki fragment ligation by ChVLig. While PCNA failed to unload in the Cdc9-degraded cells carrying empty vector, this accumulation of PCNA on chromatin was substantially reduced when ChVLig is overexpressed (Figures 6G and 6H). These results indicate that PCNA can be unloaded following Okazaki fragment ligation mediated by ChVLig. Indeed, in these experiments the kinetics of PCNA unloading closely mirror those of Okazaki fragment ligation: PCNA is unloaded by 70 min in the presence of wild-type Cdc9 (Figure S6D), but although at 70 min replication appears almost finished in Cdc9-degraded cells overexpressing ChVLig (Figure 6D), significant amounts of PCNA remain on chromatin (compare Figure 6G with Figure S6D). This slower unloading of PCNA probably reflects incomplete Okazaki fragment ligation by ChVLig at the 70-min time point (Figures 6E and 6F). Taken together, these results imply that *Chlorella* virus DNA ligase can ligate a large fraction Okazaki fragments and thereby facilitate partial unloading of PCNA from chromatin.

### Elg1 Is Required to Unload PCNA Efficiently following Okazaki Fragment Ligation by *Chlorella* Virus DNA Ligase

**Figure 7:**
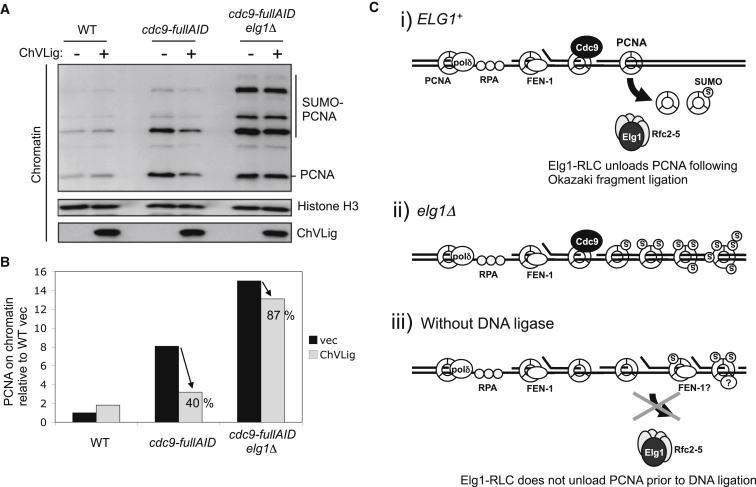
Elg1 Is Required for Efficient PCNA Unloading following Okazaki Fragment Ligation by *Chlorella* Virus DNA Ligase (A) Chromatin-enriched fractions prepared from the indicated strains with/without *Chlorella* virus DNA ligase (ChVLig: +/−). Expression of ChVLig, cell-cycle synchronization, and Cdc9 degradation were performed as for Figure 6C. Cells were collected in S phase (40 min after release). Chromatin-bound proteins were detected by western blotting. (B) Quantification of chromatin-bound PCNA in (A), expressed relative to wild-type (WT) carrying empty vector i.e., lane 1 in (A). Expression of ChVLig reduced chromatin-bound PCNA to 40% in *cdc9-fullAID* cells, but only to 87% in *cdc9-fullAID elg1Δ* cells. (C) Model of PCNA unloading by the Elg1-RLC. (i) In an *ELG1*^+^ strain, PCNA and SUMO-PCNA are unloaded by the Elg1-RLC from the lagging strand following Okazaki fragment ligation. (ii) In an *elg1*Δ mutant, PCNA is retained on chromatin. (iii) Removal of the DNA ligase Cdc9 causes PCNA retention on chromatin, as Elg1-RLC does not unload PCNA prior to DNA ligation. This failure of PCNA unloading could potentially be caused by continuing presence of other PCNA interacting partners (such as DNA polymerase δ or FEN-1), which might interfere with access to PCNA by Elg1. See also Figure S7.

We then tested if PCNA unloading following Okazaki fragment ligation by ChVLig requires Elg1. We collected wild-type, *cdc9-degron*, or *cdc9-degron elg1Δ* cells, with or without overexpressed ChVLig, at a mid-S phase time point, and we examined chromatin-bound PCNA (Figure 7A). As in Figure 6G, overexpression of ChVLig in *cdc9-degron* cells in the presence of Elg1 reduced PCNA accumulation on chromatin (Figure 7A, third and fourth lanes). Overexpression of ChVLig in *cdc9-degron* cells in the absence of Elg1 also led to some reduction of PCNA on chromatin (Figure 7A, sixth lane), but to a lesser extent than in the presence of Elg1 (overexpression of ChVLig reducing PCNA accumulation on chromatin to 40% in the presence of Elg1 and 87% in the absence of Elg1; Figure 7B). We repeated this experiment and observed similar Elg1-dependent PCNA unloading when ChVLig is overexpressed in *cdc9-degron* cells (Figure S7). These results indicate that Elg1 is required for efficient and timely unloading of PCNA following Okazaki fragment ligation by ChVLig. As previously described (Kubota et al., 2013b), in the absence of Elg1, a slower “backup” unloading mechanism eventually enables removal of PCNA, the molecular nature of which is unclear. The effect of expressing ChVLig on PCNA retention in the *elg1Δ* mutant suggests that Okazaki fragment ligation may also assist backup PCNA unloading, but less strongly than it promotes unloading by the Elg1-RLC.

Taken together, the results we present establish that Okazaki fragment ligation is a key step required to enable effective and timely PCNA unloading by Elg1-RLC during DNA replication.

## Discussion

We have tested where and when Elg1-RLC unloads PCNA from DNA during DNA replication. Using the ChIP-seq method, we observed that PCNA is retained on chromosomes in the wake of replication forks in an *elg1Δ* mutant. We show that Elg1-RLC fails to unload PCNA prior to Okazaki fragment ligation by Cdc9, and that Okazaki fragment ligation by the exogenous DNA ligase ChVLig promotes PCNA unloading by Elg1-RLC. On the basis of these observations, we propose that Elg1-RLC acts as a general PCNA unloader during DNA replication, removing PCNA from DNA following Okazaki fragment ligation on the lagging strand (Figure 7C).

Using ChIP in well-synchronized cultures, we found that the accumulation of PCNA on chromatin in the absence of Elg1 is primarily caused by PCNA retention on chromosomes following replication fork passage (Figure 2). Although the *elg1Δ* mutant shows a cohesion defect (Maradeo and Skibbens, 2009; Parnas et al., 2009), we observed no clear correlation between PCNA accumulation and cohesion sites (Figure S3; data are available at http://www.iam.u-tokyo.ac.jp/chromosomeinformatics/Supplementary%20Material.html). It has been suggested that a specialized mode of PCNA unloading contributes to cohesion establishment (Bylund and Burgers, 2005), but our results do not support the possibility that Elg1-RLC acts specifically at sites of cohesion. Our results do not exclude the possibility that levels of PCNA accumulation occurring as replication forks transit cohesion sites lead to the observed cohesion defect of the *elg1Δ* mutant.

To support DNA synthesis by Polδ, PCNA is proposed to be loaded repeatedly on the lagging strand at initiation of each Okazaki fragment, then unloaded after completion of Okazaki fragment processing. Our results suggest that Okazaki fragment ligation must occur prior to Elg1-dependent PCNA unloading, illustrated by the fact that PCNA accumulates on chromatin in ligase-null *cdc9* mutants and in cells with Cdc9 degraded. We found that expression of the exogenous DNA ligase *Chlorella* virus DNA ligase in place of Cdc9 promotes PCNA unloading by Elg1-RLC. Consistent with our demonstration that Elg1-RLC unloads PCNA from the lagging strand following Okazaki fragment ligation, a recent paper from the Zhang group used strand-specific analysis of PCNA binding to show that in a blocked S phase PCNA is normally removed from the lagging strand, but in an *elg1Δ* mutant, PCNA instead accumulates on the lagging strand (Yu et al., 2014).

Why does PCNA unloading by Elg1-RLC require Okazaki fragment ligation? One possibility is that Okazaki fragment ligation causes removal of other components that may compete with Elg1-RLC for interaction with PCNA. PCNA is involved in multiple steps of lagging-strand processing, acting as a recruitment platform for Polδ, FEN-1, and CAF-1 as well as for Cdc9. Through these interactions, PCNA ensures processive strand extension by Polδ and recruits FEN-1 for cleavage of the flap structure. Both of these steps precede Okazaki fragment ligation, as may the action of CAF-1, which has been proposed to deposit histone H3-H4 behind PCNA prior to Okazaki fragment ligation (Smith and Whitehouse, 2012). If so, Okazaki fragment ligation may be the last step of lagging-strand processing, after which PCNA will be left on double-stranded DNA lacking any 3′ end and also presumably lacking the above PCNA interactors. We suspect that such “free” PCNA may be a preferential target of Elg1-RLC (Figure 7Ci). PCNA interactors like Polδ, FEN-1, and Cdc9 could compete quite effectively with Elg1-RLC for PCNA interaction (Figure 7Ciii), since Elg1-RLC probably interacts with all three subunits of PCNA, on the basis of its structural resemblance to RFC. In cells lacking Cdc9 activity, Polδ and/or FEN-1 may continue to interact with PCNA at 3′ DNA ends, preventing Elg1-RLC from engaging PCNA. Single-strand binding protein RPA could also potentially inhibit access of Elg1-RLC to PCNA, which might explain why no PCNA unloading by Elg1-RLC was detected in vitro, in experiments that used a substrate with PCNA loaded at the 3′ end of an RPA-coated primer template junction (Bylund and Burgers, 2005).

Does PCNA modification affect unloading by Elg1-RLC? Elg1-RLC preferentially interacts with SUMOylated PCNA (Parnas et al., 2010). The effects of PCNA modification on unloading by Elg1-RLC have not been tested in detail, and Elg1-RLC may preferentially unload SUMOylated PCNA (although SUMOylation is not essential for Elg1-mediated PCNA unloading; Kubota et al., 2013b; Yu et al., 2014). We observed an increased proportion of di- or poly-SUMOylated PCNA on chromatin in an *elg1Δ* mutant compared to Cdc9-degraded cells (Figures 3C and 4B). This difference could potentially reflect differential ability of modified PCNA to be unloaded by Elg1-RLC—for example, unloading of di- or poly-SUMOylated PCNA by Elg1-RLC might occur even *without* preceding Okazaki fragment ligation, if for example Elg1-RLC binds di- or poly-SUMOylated PCNA more strongly than do other PCNA interactors.

In summary, the results presented here imply that Elg1-RLC acts as a general PCNA unloader following Okazaki fragment ligation. We suggest possibilities above for why Okazaki fragment ligation must precede PCNA unloading, possibilities that will be testable with the development of an in vitro unloading assay using defined components. It will also be interesting to understand whether human Elg1-RLC also unloads PCNA following Okazaki fragment ligation.

## Experimental Procedures

### Yeast Strains

*S. cerevisiae* strains used are listed in Table S1. Epitope and AID tagging and gene disruptions were carried out using standard PCR-based gene-insertion methods (Longtine et al., 1998; Nishimura et al., 2009). Tagged and disrupted alleles were confirmed by PCR. To construct strains expressing *POL30-3myc* as a second copy, a fragment spanning the tagged *POL30* gene of TKY212 (524 bp upstream of *POL30* to 3myc-kanMX cassette) was amplified by PCR and inserted into the *his3* locus by homologous recombination. The sequence of this *his3Δ::P*_*POL30*_*-POL30-3myc::kanMX* allele was directly confirmed.

### Plasmid Construction

Plasmids encoding *CDC9*, *cdc9* mutants, and *ChVLig* (pRS313*-P*_*CDC9*_*-CDC9,* pRS313*-P*_*CDC9*_*-cdc9-NΔ60,* pRS313*-P*_*CDC9*_*-cdc9-K419A*, pRS313*-P*_*CDC9*_*-cdc9-K598A*, pRS423-*P*_*GAL1–10*_-*ChVLig-3HA*) were as described previously (Nguyen et al., 2013). Plasmid pRS305*- P*_*GAL1–10*_*-POL30-3myc* was constructed by cloning a fragment containing *POL30-3myc* (amplified by PCR from genomic DNA prepared from TKY212) into pRS305-*P*_*GAL1–10*_, using In-Fusion HD cloning (Takara Clontech). Similarly, plasmids pRS303*-P*_*GAL1–10*_*-ELG1-13Myc,* pRS305*-P*_*GAL1–10*_*-CDC9-3HA*, pRS305*-P*_*GAL1–10*_*-cdc9-NΔ60-3HA*, pRS305*-P*_*GAL1–10*_*-cdc9-K419A-3HA*, and pRS305*-P*_*GAL1–10*_*-cdc9-K598A-3HA* were constructed by In-Fusion HD cloning.

### Synchronization and Induction of Protein Degradation

Synchronization of cells, induction of protein degradation, and sample collection were performed as shown in the experimental outline of each figure and as described previously (Kubota et al., 2013b).

### Preparation of Whole-Cell Extracts and Chromatin-Enriched Fractions

For preparation of whole-cell extracts (WCEs), cells were sampled at indicated time points, washed once with water, incubated in 0.1 M NaOH for 5 min at room temperature, spun down, and resuspended in SDS sample buffer.

Chromatin-enriched fractions were prepared as described previously (Kubota et al., 2011, 2012) with the following modifications: we increased the concentration of sorbitol in spheroplasting buffer and ice-cold wash buffer to 0.8 M, and we omitted spinning of spheroplasts through 7.5% Ficoll-sorbitol cushion. To summarize briefly, spheroplasted cells were dropped into buffer containing 18% Ficoll (at this stage, it was confirmed microscopically that the cytoplasmic membranes were lysed but nuclei were intact), and unbroken cells were removed by low-speed spin (5,000 × *g* for 5 min). Nuclei were then pelleted by a high-speed spin (16,100 × *g* for 20 min) and lysed in buffer containing 0.25% Triton X-100. A chromatin pellet was then prepared by centrifugation through a 30% sucrose cushion.

### Western Blotting and Quantification

Western blotting and quantification were performed as described previously (Kubota et al., 2011). Antibodies used for western blotting were mouse monoclonal anti-PCNA (ab70472, Abcam), rabbit polyclonal anti-histone H3 (ab46765, Abcam), rabbit polyclonal anti-myc (ab9106, Abcam), and mouse monoclonal anti-hemagglutinin (anti-HA) (HA.11 clone 16B12, Covance) antibodies.

### ChIP-Seq Analysis

*cdc7-1* and *cdc7-1 elg1Δ* cells carrying the additional copy of *POL30-3myc* (TKY245 and TKY247) were collected at G1 phase and early and mid-S phase time points. Cells were fixed with 1% formaldehyde at room temperature for 20 min and then at 4°C overnight. The fixed cells were washed in ice-cold PBS three times and stored at −20°C. ChIP was performed as previously described (De Piccoli et al., 2012; Katou et al., 2003). DNA from WCEs and ChIP fractions was sequenced on the Hiseq 2500 to generate single-end 50-bp reads. The reads were mapped onto the reference genome obtained from *Saccharomyces* Genome Database (http://www.yeastgenome.org/) using Bowtie (Langmead et al., 2009), allowing two mismatches and multiply mapped reads (-n2 -k1 option). For normalization and visualization of ChIP-seq data, we used DROMPA (Nakato et al., 2013). To minimize bias in high-throughput sequencing, we focused the enrichment of the number of sequence reads from the ChIP fraction against that of the corresponding WCE fraction at each 100-bp window. To visualize the comparative enrichment of PCNA signals on replicated sites compared to unreplicated sites, ChIP-seq signals were normalized to give unreplicated regions a value of 1 (e.g., regions close to 360 kb in Figure 2E).

To perform meta-analysis, early and late origins were designated as described previously (Crabbé et al., 2010) (i.e., early origins as those that fire in wild-type cells in the presence of the replication inhibitor hydroxyurea [HU], and late origins as those that do not fire in wild-type but fire in the checkpoint-deficient mutant *rad53-11* in HU). Only “confirmed” origins referenced in the database OriDB (Nieduszynski et al., 2007) were included (i.e., “dubious” and “likely” origins were removed). ChIP-seq data were aligned to origins, and the median enrichment in PCNA signals was plotted.

### In Vitro PCNA Unloading Assay by Lysing Nuclei in Cell Extracts

The in vitro PCNA unloading assay was performed as described previously (Supplemental Experimental Procedures in Kubota et al., 2013b). Briefly, nuclei isolated from *elg1Δ* or *cdc9-degron* cells (TKY195 or TKY243) in S phase were lysed in soluble cell extracts from cells expressing PCNA-3myc but lacking Elg1 and Cdc9 (TKY305: *elg1Δ cdc9-degron POL30-3myc*) or cells expressing PCNA-3myc and overexpressing Elg1 but lacking Cdc9 (TKY272: *GALpr-ELG1-13myc cdc9-degron POL30-3myc*), as illustrated in Figure 4A. After incubating at 30°C for 10 min, a chromatin-enriched fraction was prepared. Chromatin-associated PCNA was examined by western blotting. For the “No treatment” control sample, nuclei were lysed in buffer (50 mM HEPES/KOH [pH 7.5], 50 mM potassium acetate, 10 mM magnesium acetate, 1 mM DTT, 10% glycerol, 0.5% Triton X-100, 2 mM NaF, 2 mM β-glycerophosphate, 1 mM ATP, 0.5 mM spermidine, 1 × complete protease inhibitor cocktail (Roche), 1% Protease Inhibitor Cocktail [Sigma P8215], and 1 mM PMSF) and a chromatin-enriched fraction then prepared.

### Immunoprecipitation

Immunoprecipitation was performed as described previously (Kubota et al., 2011). Briefly, ∼1 × 10^8^ cells grown in the presence of galactose were spheroplasted and lysed in 600 μl low-salt buffer (which contains 50 mM potassium acetate). Soluble lysates were prepared by centrifugation following DNase I treatment and then used for immunoprecipitation with anti-HA antibody (2 μg, HA.11 clone 16B12) coupled to Dynabeads Protein G. PCNA was first eluted from the beads in 20 μl of the buffer containing 1 M NaCl (to minimize simultaneous elution of the antibody, as the antibody light chain overlaps with PCNA in western blotting). HA-tagged proteins were then eluted in 50 μl 1 × SDS sample buffer.

### Okazaki Fragment Detection Assay

Okazaki fragments were labeled and detected as described previously (Smith and Whitehouse, 2012).

## Author Contributions

T.K. performed most experiments; Y.K. performed ChIP-seq experiments; R.N. and T.K. analyzed ChIP-seq data; K.S. advised on the design and analysis of ChIP-seq experiments; and T.K. and A.D.D. designed the experiments and wrote the manuscript.

## Supplemental Information

Document S1. Figures S1–S7

Table S1. Yeast Strains Used in This Study, Related to Figures 1, 2, 3, 4, 5, 6, and 7

Document S2. Article plus Supplemental Information
