# Supplementary material for: Replication-Coupled PCNA Unloading by the Elg1 Complex Occurs Genome-wide and Requires Okazaki Fragment Ligation
Source: Cell Rep. 2015 Jul 23;12(5):774–87. doi: 10.1016/j.celrep.2015.06.066 (PMC4534484; doi:10.1016/j.celrep.2015.06.066)
Supplement: Document S1. Figures S1–S7 [file mmc1.pdf]

Cell Reports

Supplemental Information

Replication-Coupled PCNA Unloading by the Elg1 Complex Occurs Genome-wide and Requires Okazaki Fragment Ligation

Takashi Kubota, Yuki Katou, Ryuichiro Nakato, Katsuhiko Shirahige, and Anne D. Donaldson

Fig. S1

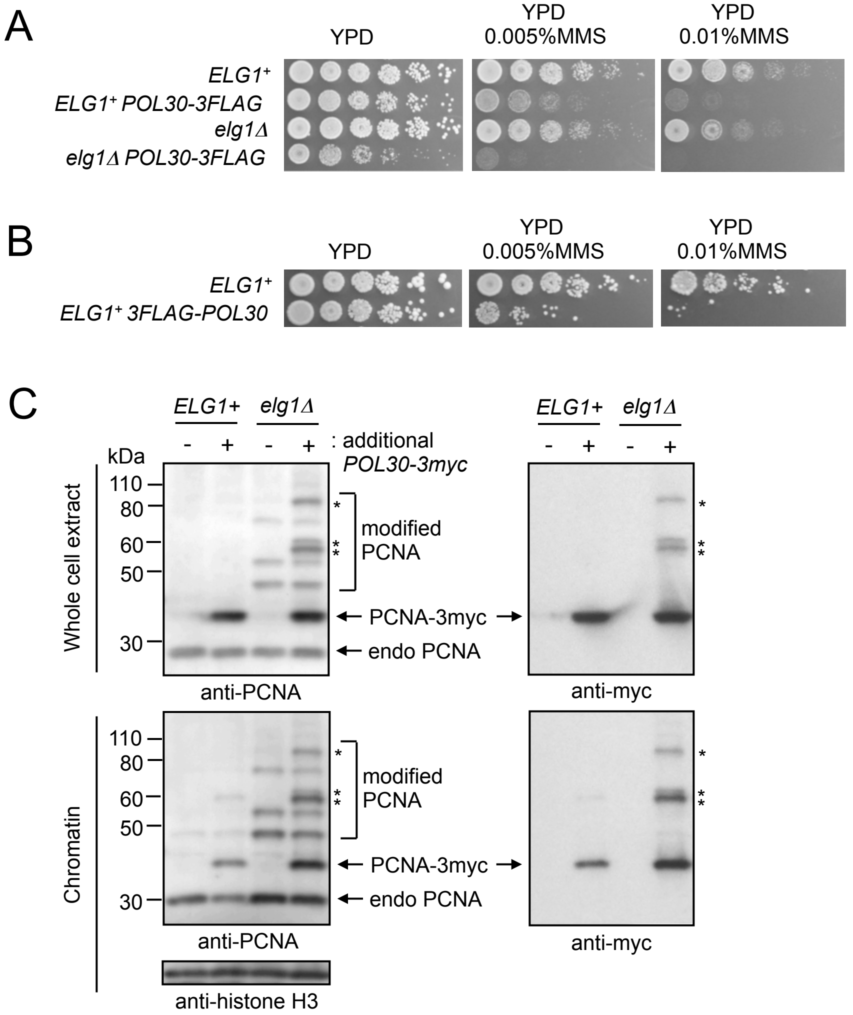
A
YPD
YPD 0.005%MMS
YPD 0.01%MMS
ELG1+
ELG1+ POL30-3FLAG
elg1Δ
elg1Δ POL30-3FLAG
B
YPD
YPD 0.005%MMS
YPD 0.01%MMS
ELG1+
ELG1+ 3FLAG-POL30
C
ELG1+
elg1Δ
- + - +
: additional POL30-3myc
kDa
110
80
60
50
30
Whole cell extract
modified PCNA
PCNA-3myc
endo PCNA
anti-PCNA
anti-myc
Chromatin
anti-histone H3

Fig. S2

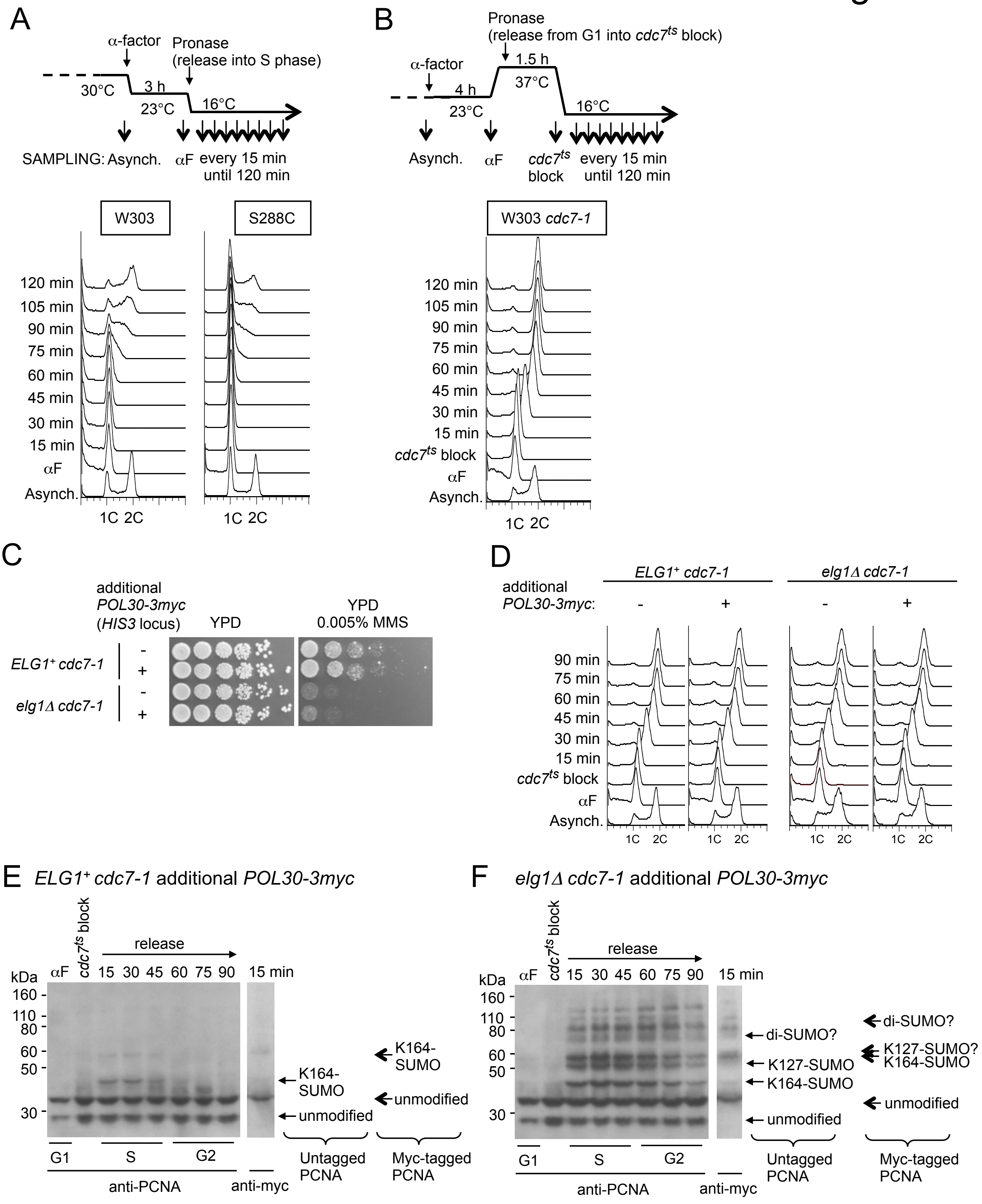
A
α-factor
Pronase
(release into S phase)
30°C
3 h
23°C
16°C
SAMPLING: Asynch.
αF
every 15 min
until 120 min
W303
S288C
120 min
105 min
90 min
75 min
60 min
45 min
30 min
15 min
αF
Asynch.
1C 2C
B
Pronase
(release from G1 into cdc7ts block)
α-factor
1.5 h
4 h
37°C
23°C
16°C
Asynch.
αF
cdc7ts block
every 15 min
until 120 min
W303 cdc7-1
cdc7ts block
C
additional
POL30-3myc
(HIS3 locus)
YPD
YPD
0.005% MMS
ELG1+ cdc7-1
elg1Δ cdc7-1
D
ELG1+ cdc7-1
elg1Δ cdc7-1
additional
POL30-3myc:
E
ELG1+ cdc7-1 additional POL30-3myc
release
kDa
160
110
80
60
50
30
15 30 45 60 75 90
15 min
K164-SUMO
unmodified
G1
S
G2
anti-PCNA
anti-myc
Untagged PCNA
Myc-tagged PCNA
F
elg1Δ cdc7-1 additional POL30-3myc
di-SUMO?
K127-SUMO
K164-SUMO
K127-SUMO?

Chromosome XI

Fig. S3

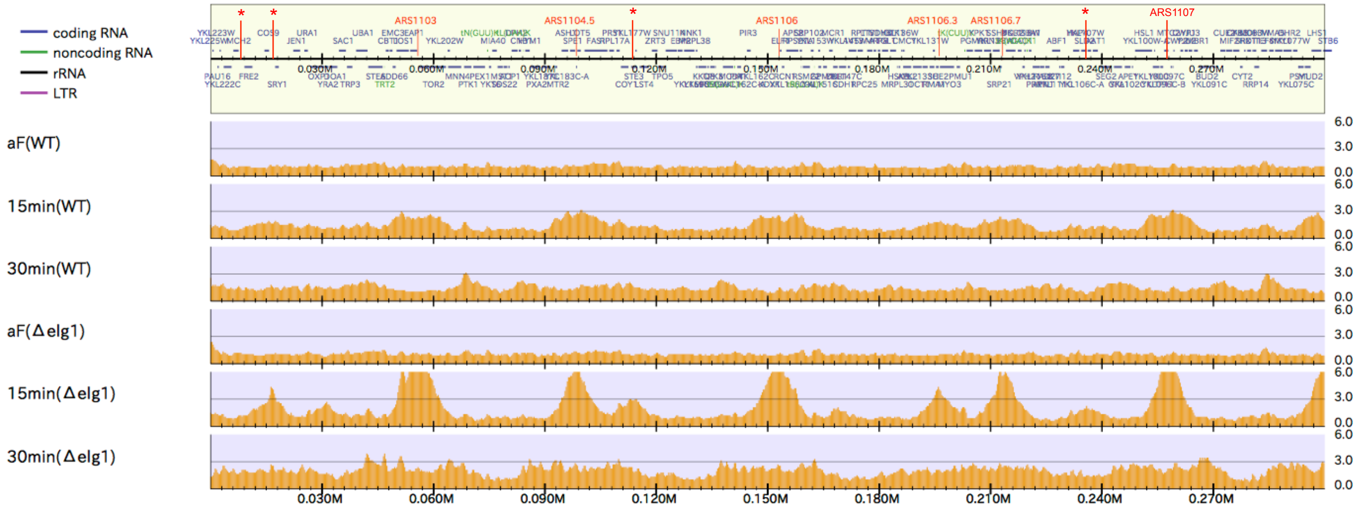

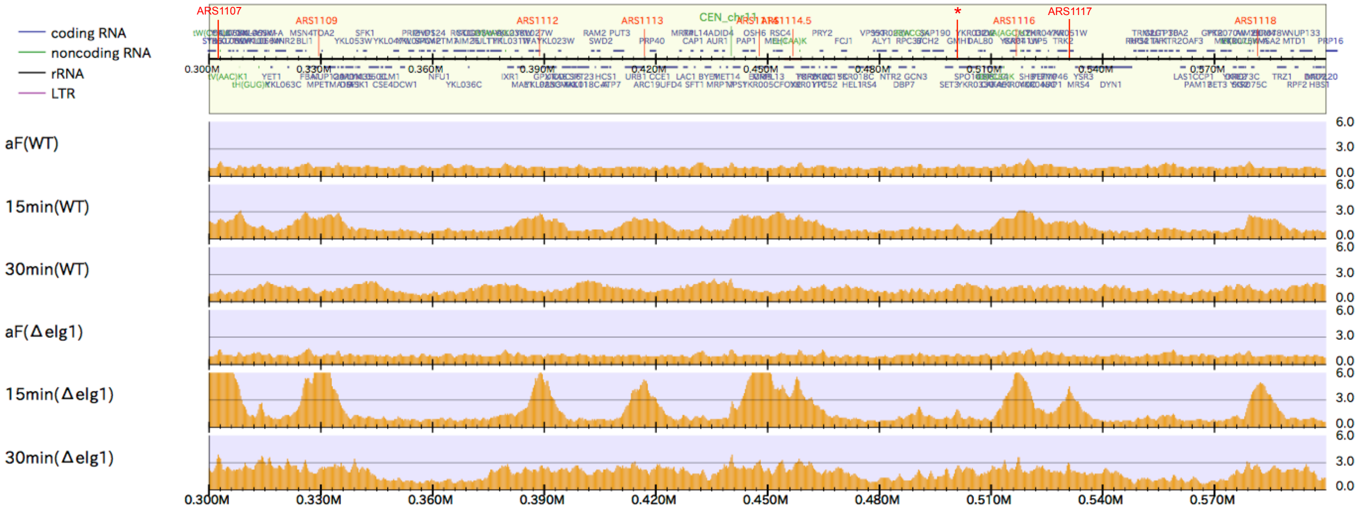

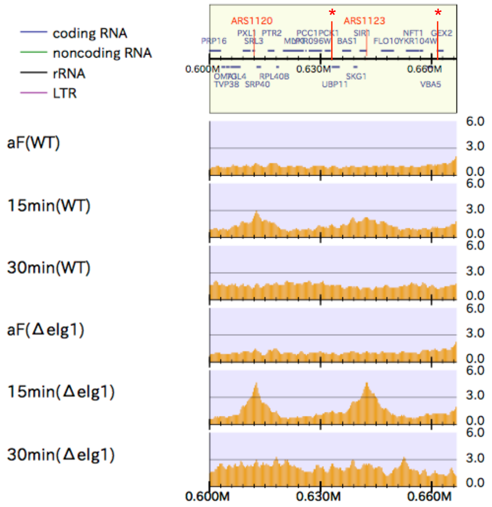

Fig. S4

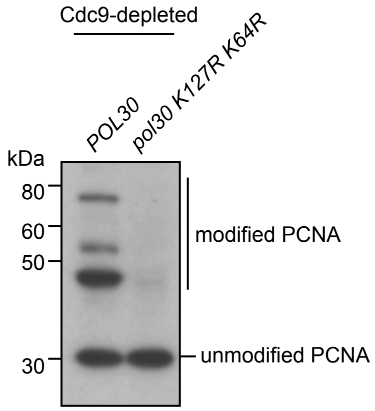
Cdc9-depleted
POL30
pol30 K127R K64R
kDa
80
60
50
30
modified PCNA
unmodified PCNA

Fig. S5

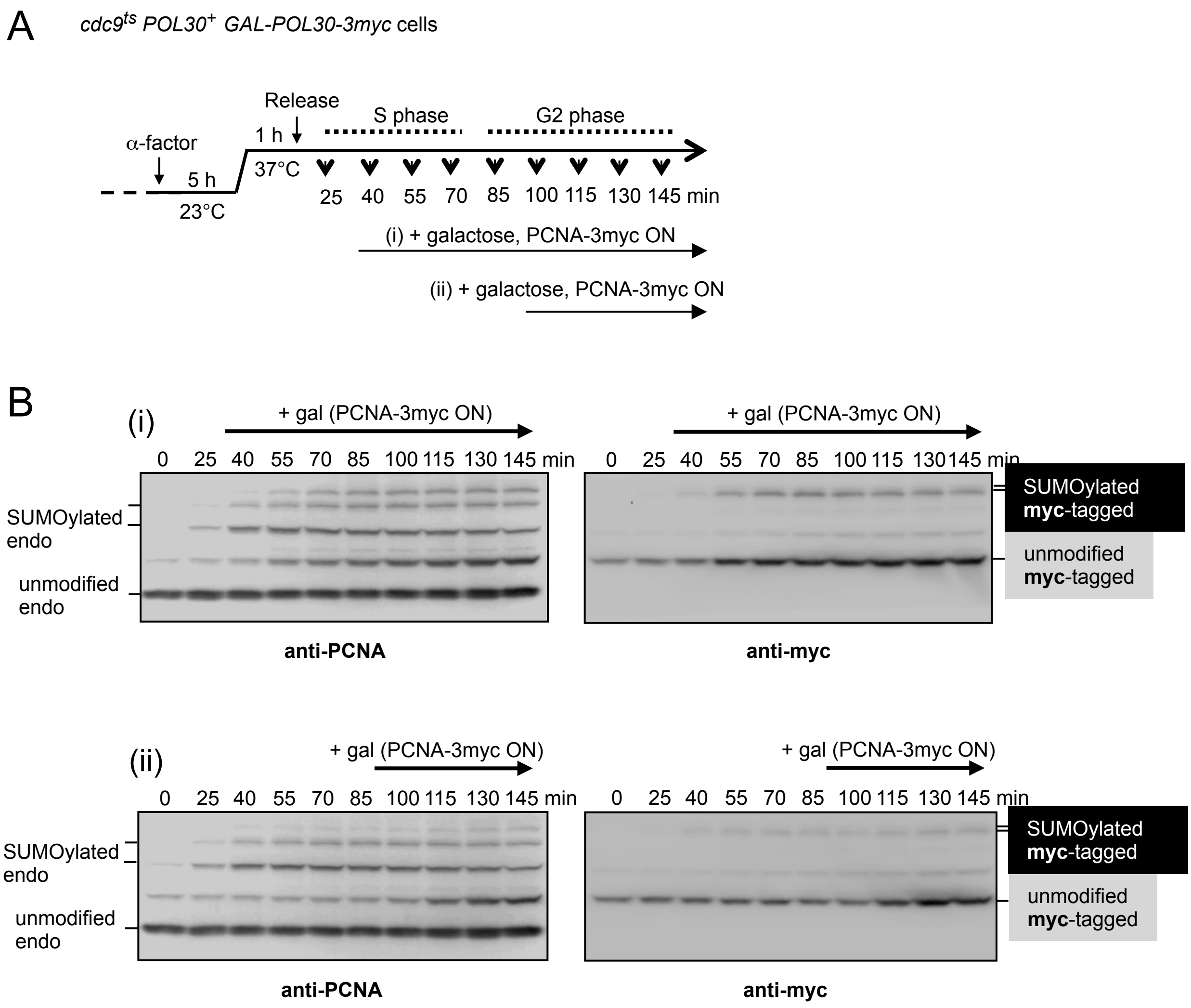
A
cdc9ts POL30+ GAL-POL30-3myc cells
Release
S phase
G2 phase
1 h
α-factor
37°C
5 h
23°C
25 40 55 70 85 100 115 130 145 min
(i) + galactose, PCNA-3myc ON
(ii) + galactose, PCNA-3myc ON
B
(i)
+ gal (PCNA-3myc ON)
0 25 40 55 70 85 100 115 130 145 min
SUMOylated endo
unmodified endo
SUMOylated myc-tagged
unmodified myc-tagged
anti-PCNA
anti-myc
(ii)
+ gal (PCNA-3myc ON)
0 25 40 55 70 85 100 115 130 145 min
SUMOylated endo
unmodified endo
SUMOylated myc-tagged
unmodified myc-tagged
anti-PCNA
anti-myc

Fig. S6

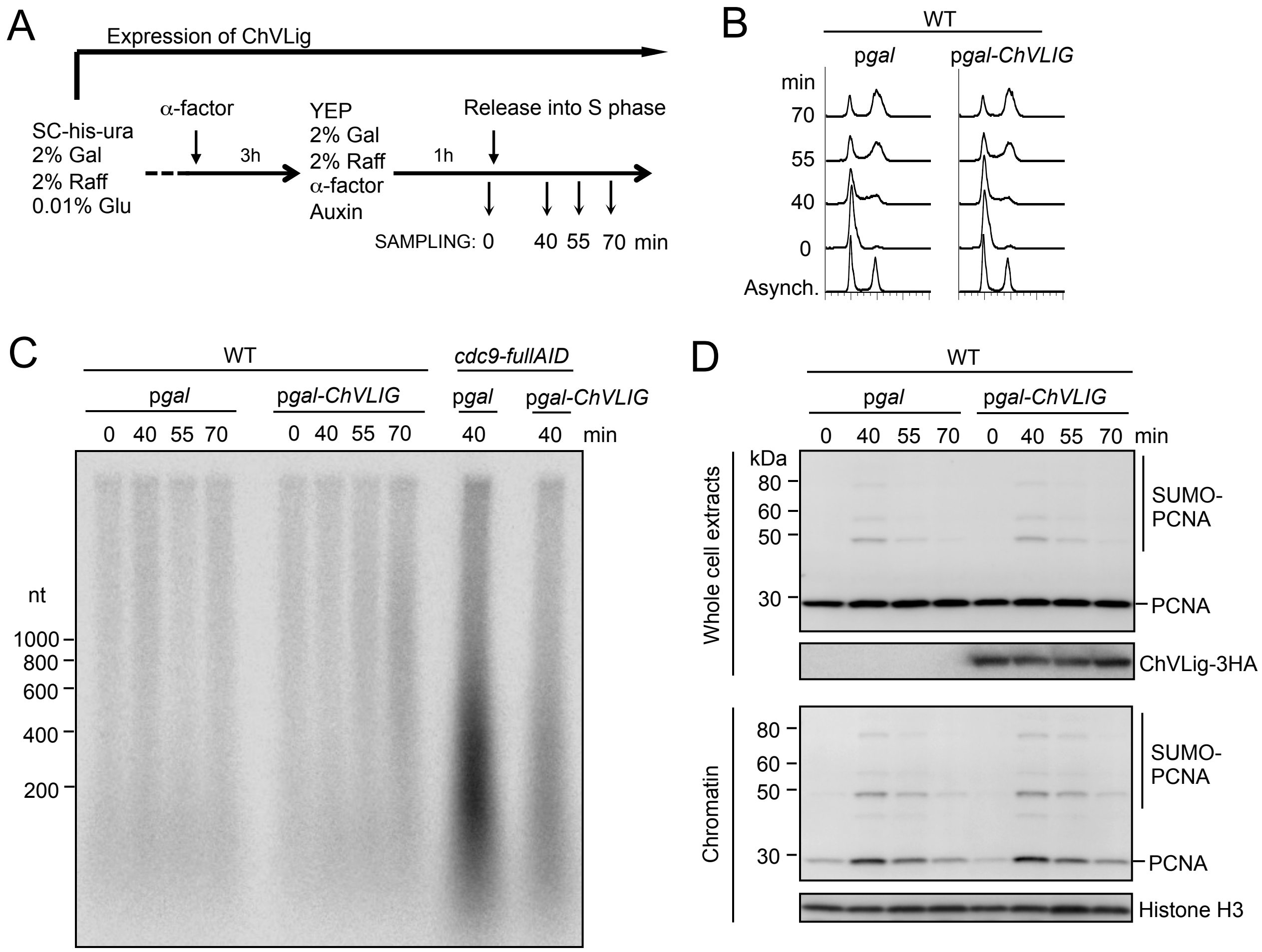
A
Expression of ChVLig
SC-his-ura
2% Gal
2% Raff
0.01% Glu
α-factor
3h
YEP
2% Gal
2% Raff
α-factor
Auxin
1h
Release into S phase
SAMPLING: 0 40 55 70 min
B
WT
pgal
pgal-ChVLIG
min
70
55
40
0
Asynch.
C
WT
cdc9-fullAID
pgal
pgal-ChVLIG
0 40 55 70
40
min
nt
1000
800
600
400
200
D
WT
pgal
pgal-ChVLIG
0 40 55 70 0 40 55 70 min
kDa
80
60
50
30
Whole cell extracts
SUMO-PCNA
PCNA
ChVLig-3HA
Chromatin
Histone H3

Fig. S7

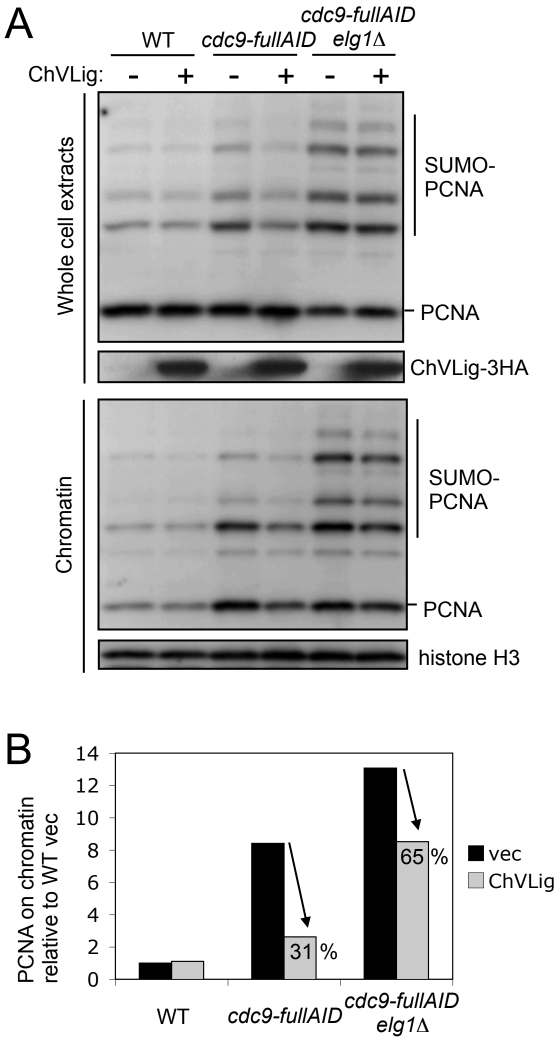
A
WT
cdc9-fullAID
cdc9-fullAID
elg1Δ
ChVLig:
-
+
-
+
-
+
Whole cell extracts
SUMO-
PCNA
PCNA
ChVLig-3HA
Chromatin
SUMO-
PCNA
PCNA
histone H3
B
PCNA on chromatin
relative to WT vec
14
12
10
8
6
4
2
0
31 %
65 %
vec
ChVLig
WT
cdc9-fullAID
cdc9-fullAID
elg1Δ

Supplemental Figure legends

Figure S1, related to main Figure 1. PCNA trimer consisting of all three FLAG-tagged subunits is not fully functional.

(A & B) Strains carrying PCNA tagged with FLAG at C-terminus (A) or N-terminus (B) show increased sensitivity to MMS, compared to untagged strains. Five-fold serial dilutions of cells plated on YPD or YPD plus MMS, incubated for 2 days at 30°C.

(C) Both myc-tagged and untagged PCNA are loaded on chromatin and, in an *elg1Δ* mutant, accumulate on chromatin. Whole cell extracts and chromatin fractions were prepared from log phase cells. Both myc-tagged and untagged PCNA were detected by anti-PCNA antibody (left), and myc-tagged PCNA bands were confirmed by re-probing with anti-myc antibody (right). Asterisks indicate modified myc-tagged PCNA. Histone H3 is loading control.

Figure S2, related to main Figure 2. Synchronization efficiency and characterisation of *cdc7-1* strains expressing both myc-tagged and untagged PCNA.

(A & B) Cell cycle synchronization of (A) W303 and S288C strains and (B) W303 *cdc7-1* strain, analysed by flow cytometry.

(C) Expression of additional PCNA-3myc has no effect on cell growth of *cdc7-1* strains. Five-fold serial dilutions of cells plated on YPD or YPD plus MMS, incubated for 3 days at 23°C.

(D) Flow cytometry analysis of strains indicated. Cell cycle synchronization performed as shown in (B). Expression of myc-tagged PCNA in addition to untagged PCNA does not affect S phase progression in the *cdc7-1* strain background.

(E & F) Both myc-tagged and untagged PCNA are normally SUMOylated, suggesting that both are loaded on DNA during S phase in (E) *ELG1*$^{+}$ and (F) *elg1*Δ cells in the *cdc7-1* mutant background. As previously reported in Kubota et al. 2013, PCNA is still SUMOylated in G2 phase in *elg1*Δ mutant, suggesting that PCNA is retained on chromatin in the absence of Elg1 even after DNA replication.

Figure S3, related to main Figure 2. Analysis of PCNA accumulation on replicated DNA in the absence of Elg1.

PCNA ChIP-seq analysis was performed as shown in main Figure 2; results for entire chromosome XI presented here. Asterisks indicate 'likely' origins listed in the OriDB database (Nieduszynski et al. 2007). Other chromosomes presented at http://www.iam.u-tokyo.ac.jp/chromosomeinformatics/Supplementary%20Material.html.

Figure S4, related to main Figure 3, Confirmation of PCNA modifications occurring upon Cdc9 degradation.

The majority of modified PCNA forms observed in Cdc9-depleted cells are not present in a *pol30 K127R K164R* mutant background.

Figure S5, related to main Figure 4, Confirmation of expression of myc-tagged PCNA in the experiments shown in Figure 4 C-D.

(A) Experimental outline (same as Figure 4C).

(B) Confirmation of identity of PCNA bands. Myc-tagged PCNA was detected using anti-myc antibody (right panels) following harsh stripping of the PCNA blots shown in main Figure 4D (repeated here, left panels). Note that quantitativity in anti-myc blots is compromised by the harsh stripping procedure.

Figure S6, related to main Figure 6. In the presence of Cdc9, expression of *Chlorella* virus DNA ligase does not affect Okazaki fragment ligation and PCNA unloading.

(A) Outline of the experiments.

(B) Flow cytometry analysis.

(C) Okazaki fragment detection assay.

(D) Neither loading nor unloading of PCNA is affected by expression of ChVLig in the presence of Cdc9. PCNA in whole cell extracts and chromatin-enriched fractions detected by western blotting with anti-PCNA antibody.

Figure S7, related to main Figure 7. Experimental repeat confirming that Elg1 is required for efficient PCNA unloading following Okazaki fragment ligation by *Chlorella* virus DNA ligase.

(A) Whole cell extracts and chromatin-enriched fractions were prepared from the indicated strains with/without *Chlorella* virus DNA ligase (ChVLig: +/-), and proteins were detected by western blotting.

(B) Quantification of the amount of chromatin-bound PCNA shown in (A), expressed relative to WT carrying empty vector (vec). Expression of ChVLig reduced chromatin-bound PCNA to 31% in *cdc9-fullAID* cells and to 65% in *cdc9-fullAID elg1Δ* cells.
